# Knockdown of AKR1C3 Promoted Sorafenib Sensitivity Through Inhibiting the Phosphorylation of AKT in Hepatocellular Carcinoma

**DOI:** 10.3389/fonc.2022.823491

**Published:** 2022-03-11

**Authors:** Jia Zheng, Zhihong Yang, Yanlei Li, Li Yang, Ruili Yao

**Affiliations:** ^1^ Department of Clinical Medicine, Tangshan Vocational and Technical College, Tangshan, China; ^2^ Department of Basic Medicine, Tangshan Vocational and Technical College, Tangshan, China; ^3^ Department of Pathology, Tianjin Medical University, Tianjin, China; ^4^ Department of Obstetrics and Gynecology, Tangshan Workers’ Hospital, Tangshan, China

**Keywords:** sorafenib, hepatocellular carcinoma, AKR1C3, Akt, drug resistance

## Abstract

**Background:**

Sorafenib, which can induce ferroptosis, is a multikinase inhibitor for enhancing survival in advanced hepatocellular carcinoma (HCC). However, a considerable challenge for the treatment of HCC is sorafenib resistance. Therefore, targeting the relationship between sorafenib resistance and ferroptosis genes may provide a novel approach for the treatment of HCC.

**Materials and Methods:**

We analyzed the gene expression and clinicopathological factors from The Cancer Genome Atlas Liver Hepatocellular Carcinoma (TCGA-LIHC), International Cancer Genome Consortium (ICGC), and Gene Expression Omnibus (GEO) databases (GSE109211/GSE62813). The statistical analysis was conducted in R. Cell proliferation was assayed by MTT, cell colony-forming assay, and wound healing assay. Immunofluorescence assay and Western blot were used to evaluate the expression of AKT.

**Results:**

Many ferroptosis-related genes were upregulated in the sorafenib-resistant group. Aldo-keto reductase 1C3 (AKR1C3) was highly expressed in sorafenib-resistant patients, and the high expression of AKR1C3 was associated with the poor prognosis of patients from the TCGA and ICGC databases. MTT and colony-forming assays showing AKR1C3 overexpression enhanced the proliferation of HCC cells and acute sorafenib resistance. Knockdown of AKR1C3 inhibited the proliferation of HCC cells and increased the drug sensitivity of sorafenib. Immunofluorescence assay and Western blot proved that AKR1C3 promoted the phosphorylation of AKT.

**Conclusion:**

AKR1C3 can induce sorafenib resistance through promoting the phosphorylation of AKT in HCC. AKR1C3 inhibitors may be used in conjunction with sorafenib to become a better therapeutic target for HCC.

## Introduction

According to statistics from the International Cancer Research Center, hepatocellular carcinoma (HCC) is one of the most common malignant tumors and has a high death rate around the world (1). It progresses rapidly and has a poor prognosis. Chemotherapy is still the first treatment option for advanced HCC (2, 3). However, drug resistance often leads to failure of chemotherapy in HCC patients. Further exploration of the molecular mechanism is essential for the discovery of new chemotherapy drugs.

Ferroptosis is a kind of programmed necrosis, which is mainly caused by lipid peroxidation outside the mitochondria and the increase of ferroptosis-dependent ROS. Abnormal iron metabolism and the imbalance of the two main redox systems (lipid peroxidation and thiols) are the main stimulus factors for the production of ROS. Ferroptosis is one of the basic mechanisms of sorafenib in the treatment of HCC. Many factors related to ferroptosis have been shown to be related to liver cancer (4). Retinoblastoma (RB) protein-deficient HCC cells have a two to three times higher mortality rate than cells with normal levels of RB protein. The susceptibility of RB protein-inactivated HCC to ferroptosis is due to the increase in the concentration of reactive oxygen species in the mitochondria, which increases the cells’ oxidative stress response (5). Metallothionein-1g (MT-1G) is a new type of negative regulator of ferroptosis in hepatocellular carcinoma. MT-1G gene knockdown increases sorafenib-induced ferroptosis (6).

Aldo-keto reductase 1C3 (AKR1C3) is also known as a member of the human aldo-keto reductase family (7). The human AKR1C family is composed of four enzymes, AKR1C1–4, and AKR1C3, a monomeric, cytosolic, NAD(P) (H)-dependent oxidoreductase, is expressed in the prostate, adrenals, breast, and uterus (8, 9). Many studies have demonstrated that AKR1C3 promoted the metastasis of castration-resistant prostate cancer (10) and colorectal cancer (11). Besides, the role of AKR1C3 in many types of treatment resistance was discovered. Pharmacologic inhibition of AKR1C3 increased cellular doxorubicin content and restored drug DNA binding, cytotoxicity, and subcellular localization (12). AKR1C3 is highly expressed in metastatic and recurrent prostate cancer and in enzalutamide-resistant prostate xenograft tumors. Inhibition of AKR1C3 enzymatic activity resulted in significant inhibition of enzalutamide-resistant tumor growth (13). AKR1C3 inhibitors can overcome abiraterone resistance by reducing endocrine androgen levels and reducing AR transcription activity (14). AKR1C3 mediated doxorubicin resistance through activation of the anti-apoptosis PTEN/Akt pathway *via* PTEN loss (15). AKR1C3 is overexpressed in acute myeloid leukemia and T-cell acute lymphoblastic leukemia (16). The main mechanism of action of AKR1C3 is related to ROS production and oxidative stress signaling pathway Nrf2/antioxidant response element genes (17). Increasing evidence indicates that AKR1C3 expression is a prognostic factor for tumor progression and drug resistance in a variety of malignancies. AKR1C3 inducing sorafenib resistance in hepatocellular carcinoma remains unclear.

Through bioinformatics analysis, our study found the relationship between AKR1C3 and sorafenib resistance and further proved that AKR1C3 downregulation can significantly increase the sensitivity of liver cancer cells to sorafenib. This regulatory effect is likely to be achieved through the phosphorylation of AKT.

## Methods

### Bioinformatic Analysis

The microarray datasets GSE109211 (18) and GSE62813 (19) were downloaded from the Gene Expression Omnibus (GEO) database. GSE109211 contains the gene expression data of patients who were sensitive and resistant to sorafenib (21 sorafenib treatment responders and 46 non-responders). The raw data were standardized and analyzed by the R package “limma” from the Bioconductor project. RNA with |log2 fold change (FC)| >1.5 and *P*-value <0.05 is considered a differentially expressed gene (DEG). The online website DAVID (http://david-d.ncifcrf.gov/) was used for gene ontology annotation and KEGG pathway enrichment analysis of DEG. One hundred and twenty-one ferroptosis-related genes are from the website FerrDb. FerrDb-DEG was selected with |log2 multiple change (FC)| >1.5 and *P*-value <0.05. The data were all visualized by the R package “ggplot2.” The expression of AKR1C3 in a variety of tumor tissues was validated using the Human Protein Atlas (HPA) database.

### Plasmid Construction

Overexpression plasmids for AKR1C3 were obtained by cloning the amplified cDNA into pcDNA3.1 vectors (V79020, Invitrogen, San Diego, USA) and were verified by DNA sequencing (Tsingke, Beijing, China). Short interference RNAs (shRNAs) for AKR1C3 and the corresponding negative controls were purchased from GeneCopoeia (GeneCopoeia, China).

### Cell Culture and Transduction

Human hepatoma cell lines Huh7 and HepG2 were obtained from the Chinese Academy of Sciences, Shanghai Institutes for Biological Sciences (Shanghai, China) and cultured in Dulbecco’s modified Eagle’s medium (DMEM, HyClone, USA). Cells (1 × 10^5^) in six-well plates were incubated for 24 h in a serum-free medium and then underwent transduction with plasmids using Lipofectamine 2000 (Invitrogen, San Diego, USA). After transduction, puromycin (1 μg/ml) was added for the selection.

### MTT

Cell proliferation was analyzed by the 3-(4,5-dimethylthiazoleyl)-2,5-diphenyltetrazolium bromide (MTT) assay and performed according to the manufacturer’s protocol. HCC cells with different groups were seeded into 96-well plates at 3,000 cells/well and incubated for 48 h. In brief, the medium was removed and 100 µl fresh medium with 10% MTT solution inside was added to each well and incubated at 37°C for 2 h. The absorbance of the samples was measured at 450 nm.

### Colony-Forming Assay

Cells (2 × 10^4^) were seeded in six-well plates and incubated for 48 h. The medium was replaced by RPMI-1640 containing 10% serum for 5 days. After washing with cold PBS, the colonies were fixed using 4% polymethanol for 15 min and stained using 0.3% crystal violet solution for 30 min at room temperature.

### Wound Healing Assay

Cells (6 × 105/well) were inoculated into six-well plates. After the cells reached 60% confluence, wounds were created. Then, the cells were washed three times in PBS and cultured in complete medium. Phase-contrast microscopy was employed to photograph the wounded area for 0 and 48 h. The percentage of wound closure was calculated by using ImageJ software.

### Quantitative Real-Time PCR

TRIzol^®^ reagent (Invitrogen, USA) was used to extract total RNA from cancer cells. RNAs were reversely transcribed into cDNAs by PrimeScript RT reagent kit (TaKaRa, Japan). qRT-PCR was performed using SYBR Prime Script RT-PCR kit (TaKaRa, Japan), and the primer sequences were listed as follows: GAPDH forward, 5′-GGAGCGAGATCCCTCCAAAAT-3′; GAPDH reverse, 5′-GGCTGTTGTCATACTTCTCATGG-3′, AKR1C3 forward, 5′-GGGATCTCAACGAGACAAACG-3′; AKR1C3 reverse, 5′-AAAGGACTGGGTCCTCCAAGA.

### Western Blot Analysis

Cells were washed with ice-cold PBS and split with RIPA buffer. Then, cell lysis was quantified by BCA Protein Assay kit (Beijing Solarbio Science & Technology Co., Ltd., China). Twenty-microgram protein samples were subjected to 10% SDS-PAGE and transferred onto PVDF membranes. The membranes were then blocked with 5% non-fat milk for 1.5 h at room temperature. Subsequently, the membranes were incubated with primary antibodies (AKR1C3, ab209899, Abcam, USA, 1:1,000 dilution; p-AKT, #4060, Cell Signaling Technology, USA, 1:500 dilution; AKT, #9272, Cell Signaling Technology, USA, 1:1,000 dilution) overnight at 4°C. β-Actin (AC026, ABclonal, China, 1:1,000 dilution) was used as the loading control. The membranes were washed with TBST and incubated with secondary antibodies conjugated with horseradish peroxidase (HRP) at room temperature for 1 h. Bands were scanned by the enhanced chemiluminescence (ECL) detection system (Thermo Fisher Scientific Inc., Waltham, MA, USA).

### Immunofluorescence Assay

HCC cells were washed with PBS, fixed with 5% paraformaldehyde (PFA), and permeabilized in 0.1% Triton X-100. Then, the cells were blocked with 5% BSA for half an hour and incubated overnight at 4°C with p-AKT (1:50 dilution, #4060, Cell Signaling Technology, USA). On the second day, the cells were rewarmed for 30 min and washed with PBS three times. The section with the secondary antibody (Santa Cruz Biotechnology, Santa Cruz, USA) was incubated for 2 h. The nuclei were counterstained with DAPI. Laser confocal scanning microscopy was used to capture the experimental results (Leica TCS-SP5, Germany).

### Statistical Analysis

Student’s *t*-test was used to compare gene expression between sensitivity and resistance to sorafenib. Proportion differences were compared by chi-square test. The OS between different groups was compared by Kaplan–Meier analysis and log-rank test. All statistical analyses were performed using R software (version 3.5.3) or SPSS (version 23.0). If not specified above, a *P*-value of less than 0.05 is considered statistically significant.

## Results

### AKR1C3 Was Overexpressed in Sorafenib‐Resistant HCCs

**Figure 1:**
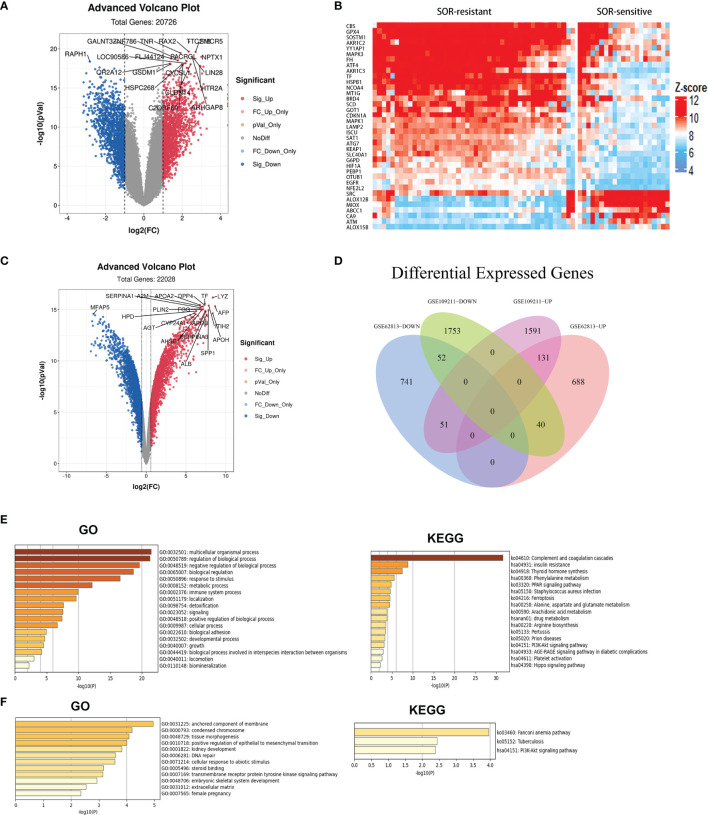
The expression of ferroptosis-related genes between sorafenib-resistant and sorafenib‐sensitive patients. **(A)** Different genes in the sorafenib-resistant group and sorafenib-sensitive group from the GEO datasets (GSE109211) were presented in the volcano map. Red dots represent significant overexpression genes and blue dots represent significantly reduced genes. **(B)** Heat maps showing 30 ferroptosis-related genes were overexpressed and 7 ferroptosis-related genes were reduced in sorafenib-resistant patients. **(C)** Different genes in the sorafenib-resistant group and sorafenib-sensitive group from the GSE62813 dataset. **(D)** The Venn diagram of different genes in the GSE109211/GSE62813 datasets. **(E)** KEGG and GO pathway enrichment of overexpression genes. **(F)** KEGG and GO pathway enrichment of genes reduced.

To investigate the molecular mechanism of sorafenib resistance in HCC, we explored the GEO database (GSE109211). We identified 1,773 genes with significant upregulation and 1,845 genes with significant downregulation in the sorafenib‐sensitive group compared with the sorafenib‐resistant group [|log2 fold change (FC)| > 1 and *P*-value < 0.05] (**Figure 1A**). Then, we next analyzed a series of ferroptosis regulators from the FerrDb database (http://www.zhounan.org/ferrdb/). Many ferroptosis-related genes were upregulated in the sorafenib-resistant group compared with those in the sorafenib-sensitive group in the GSE109211 database (**Figure 1B**). We identified 859 genes with significant overexpression and 844 genes reduced significantly in sorafenib‐resistant cells of the GSE62813 database [|log2 fold change (FC)| > 1 and *P*-value<0.05] (**Figure 1C**). We made the intersection of the two groups and found 131 genes overexpressed and 52 genes reduced significantly (**Figure 1D**). Through gene enrichment analyzed by KEGG and GO, the results revealed that the pathway associated with ferroptosis was enriched in the sorafenib‐resistant group (**Figures 1E, F**).

**Figure 2:**
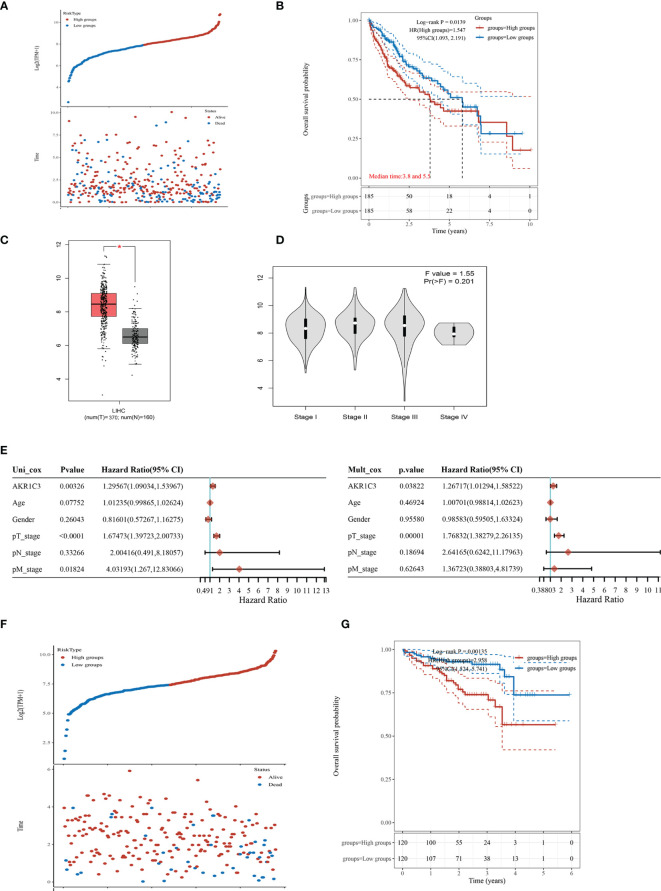
The expression of aldo-keto reductase 1C3 (AKR1C3) in liver hepatocellular carcinoma (LIHC). **(A)** AKR1C3 expression distribution and survival status based on The Cancer Genome Atlas (TCGA). **(B)** Survival analysis of AKR1C3 in LIHC based on the TCGA data. **(C)** The mRNA expression of AKR1C3 between normal and tumor tissues in TCGA. **(D)** Expression of AKR1C3 correlated with clinical stage. **(E)** Correlation between overall survival and multivariable characteristics in TCGA patients *via* Cox regression and multivariate survival model. **(F)** AKR1C3 expression distribution and survival status in the ICGC. **(G)** Survival analysis of AKR1C3 in LIHC based on the ICGC data. Data are presented as mean ± SD and are representative of three independent experiments. *P < 0.05.

We found that the mRNA level of AKR1C3 was increased obviously in the resistant HCC (**Figure 1B**). TCGA contains 370 HCC samples that included AKR1C3 expression data and various clinical characteristics. The distribution of AKR1C3 expression and the survival status of HCC patients in TCGA were shown in **Figure 2A**. The K-M survival plots showed that the group with high AKR1C3 expression had poor overall survival rates (*P*-value = 0.0139, **Figure 2B**). The expression of AKR1C3 in HCC samples was higher than that in normal liver tissue (**Figure 2C**). However, increased expression of AKR1C3 was not significantly correlated with tumor histologic grade (**Figure 2D**). Using logistic regression, univariate analysis uncovered a correlation between AKR1C3 and clinical information and pathological stage (**Figure 2E**). The distribution of AKR1C3 expression and the survival status of HCC patients from the International Cancer Genome Consortium (ICGC) database are shown in **Figure 1F**. The survival analyses of AKR1C3 in the ICGC cohort confirmed that AKR1C3 was correlated with poor OS in HCC (all adjusted *P* < 0.00135, **Figure 2G**). These results indicated that upregulation of AKR1C3 may be associated with sorafenib resistance in HCC patients and the development of HCC.

### AKR1C3 Promotes HCC Cell Proliferation

**Figure 3:**
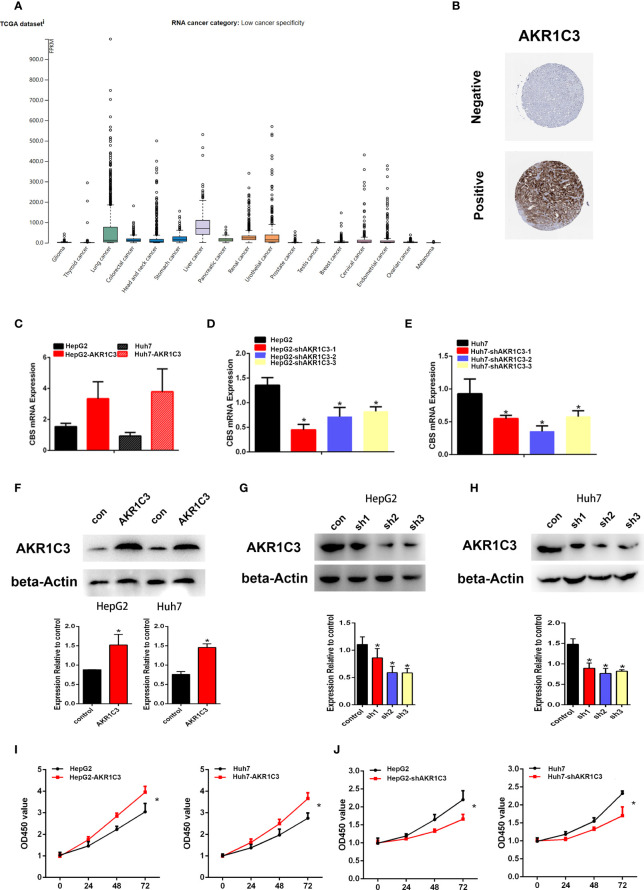
The expression of AKR1C3 in different types of tumor tissues. **(A)** The protein level of AKR1C3 in different types of tumor tissues from the HPA database. **(B)** The expression of AKR1C3 in LIHC was presented by immunohistochemistry from the HPA. **(C)** The overexpression effects of AKR1C3 in HepG2 and Huh7 cells were measured by qRT-PCR. **(D, E)** The knockdown effects of AKR1C3 in HepG2 and Huh7 cells were measured by qRT-PCR. **(F)** The overexpression effects of AKR1C3 in HepG2 and Huh7 cells were measured by Western blot. **(G, H)** The knockdown effects of AKR1C3 in HepG2 and Huh7 cells were measured by Western blot. **(I, J)** The viability of HepG2 and Huh7 cells was measured by the MTT assay. Data are presented as mean ± SD and are representative of three independent experiments. *P < 0.05.

**Figure 4:**
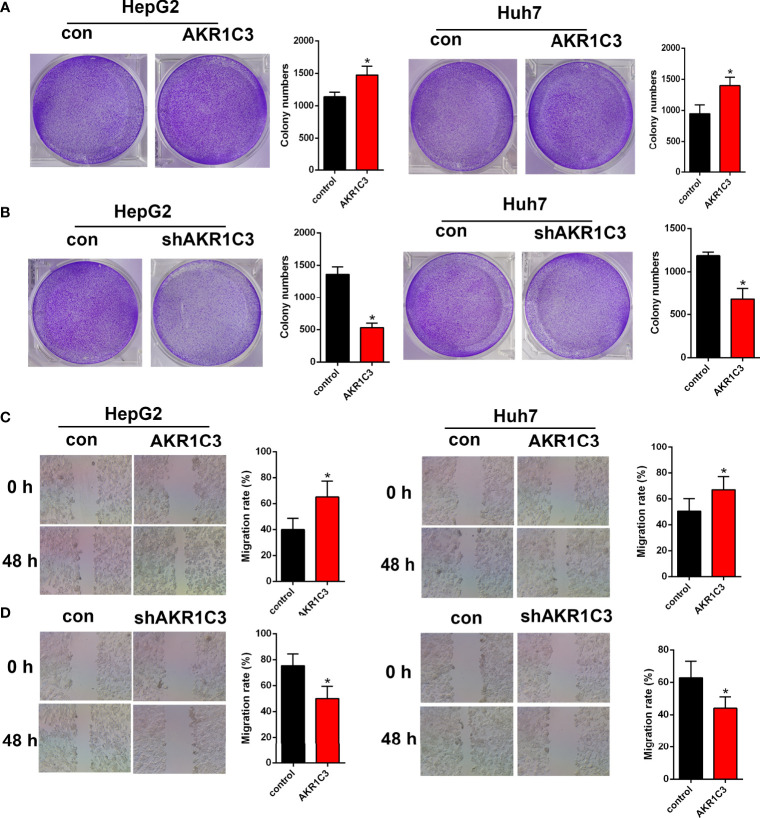
The effect of AKR1C3 on cell proliferative activity. **(A, B)** Colony formation assays in HepG2 and Huh7 cells transfected with AKR1C3 overexpression plasmids and AKR1C3 knockdown plasmids. **(C, D)** The migration of HepG2 and Huh7 cells transfected with AKR1C3 overexpression plasmids and AKR1C3 knockdown plasmids was detected by wound healing assays. Data are presented as mean ± SD and are representative of three independent experiments. *P < 0.05.

Several investigations have found that AKR1C3 in cancer cells plays an important role on a more aggressive phenotype. The expression of AKR1C3 in a variety of tumor tissues is shown in **Figure 3A**. From the HPA database, we also observed that AKR1C3 was mainly expressed in the cytoplasm and nucleus in HCC tissues (**Figure 3B**). HepG2 and Huh7 cells were transfected with AKR1C3 overexpressed plasmids, and transfection efficiency was detected by qPCR and Western blot (**Figures 3C, F**). It was found that compared with control, the overexpression of AKR1C3 increased liver cancer cell proliferation using the MTT assay (**Figure 3I**). We knockdown the AKR1C3 gene in HepG2 and Huh7 cells with three candidate lentivirus-harboring shRNAs (shRNA-1, shRNA-2, and shRNA-3). qPCR and Western blot were also used to confirm the effects of AKR1C3 knockdown on liver cancer cells, and the highest knockdown effectiveness was chosen for the subsequent experiments (**Figures 3D, E, G, H**). We noticed that, compared with the control group, the knockdown of AKR1C3 decreased the proliferation ability of HepG2 and Huh7 cells in MTT (**Figure 3J**). In the cell colony-forming assays and wound healing assays (**Figures 4A–D**), AKR1C3 overexpression in HCC cells increased the numbers of colony-forming cells and the cells’ migration ability. AKR1C3 knockdown in HCC cells suppressed the colony-forming and the migration ability of the cells. Together, these findings suggested that AKR1C3 might be mechanistically important for cell growth and migration.

### Knockdown of AKR1C3 Enhances Sorafenib Sensitivity in HCC Cells

**Figure 5:**
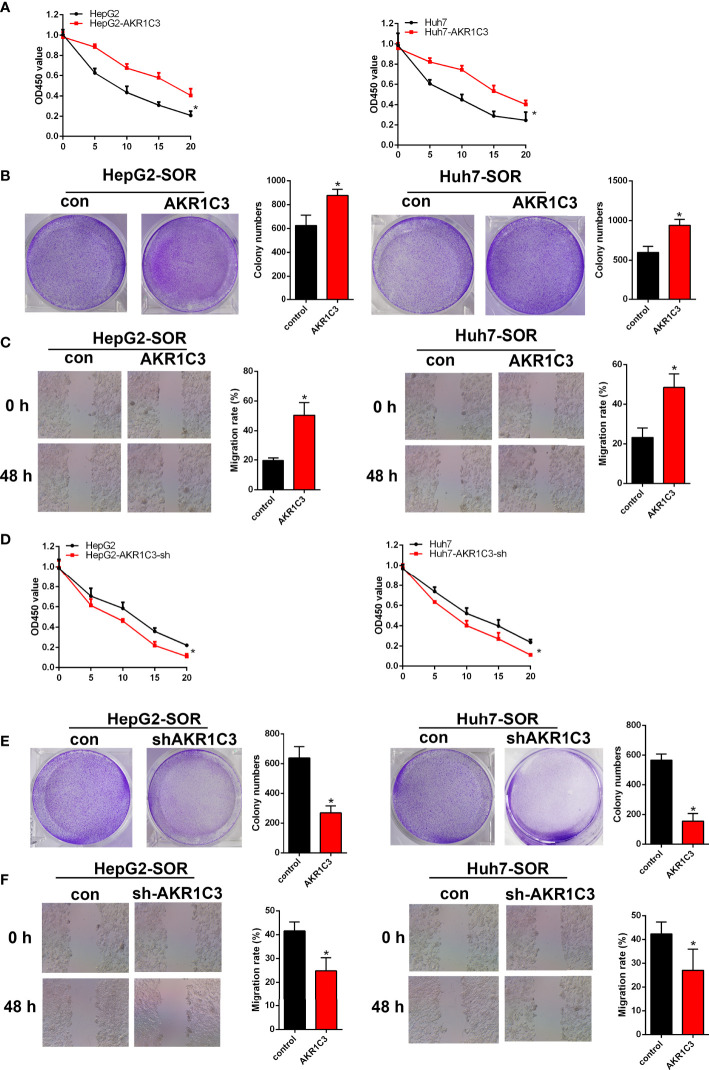
AKR1C3 enhances sorafenib resistance in HCC cells. **(A)** The viability of AKR1C3 overexpression cells after being treated with sorafenib (0, 5, 10, 15, and 20 μM) was measured by the MTT assay. **(B)** Colony formation assays in AKR1C3 overexpression cells treated with sorafenib (10 μM). **(C)** Wound healing assays in AKR1C3 overexpression cells treated with sorafenib (10 μM). **(D)** The viability of AKR1C3 knockdown cells after being treated with sorafenib (0, 5, 10, 15, and 20 μM) was measured by the MTT assay. **(E)** Colony formation assays in AKR1C3 knockdown cells treated with sorafenib (10 μM). **(F)** Wound healing assays in AKR1C3 knockdown cells treated with sorafenib (10 μM). Data are presented as mean ± SD and are representative of three independent experiments. *P < 0.05.

To evaluate whether AKR1C3 is related to sorafenib sensitivity in HCC cells, we generated a series of expression about cell proliferation. To determine this, we treated HepG2 and Huh7 cells with 0, 5, 10, 15, and 20 μM sorafenib for 48 h, and these cells included AKR1C3 overexpressed or AKR1C3 knockdown cells and the corresponding control groups. We observed that AKR1C3 overexpression significantly increased cell viability to resist sorafenib in the MTT and cell colony-forming assays (**Figures 5A, B**) and enhanced cell migration in the wound healing assays (**Figure 5C**). Meanwhile, the MTT and cell colony-forming assays showed that AKR1C3 knockdown can significantly suppress the proliferation of liver cancer cells treated with 10 μM sorafenib for 48 h (**Figures 5D, E**). In the sorafenib treatment group, downregulation of AKR1C3 significantly reduced cell migration in the wound healing assays (**Figure 5F**). Together, these findings demonstrate that knockdown of AKR1C3 in liver cancer cells induced sensitivity toward sorafenib treatment.

### AKR1C3 Influences Sorafenib Sensitivity Through AKT Phosphorylation in Liver Cancer Cells

**Figure 6:**
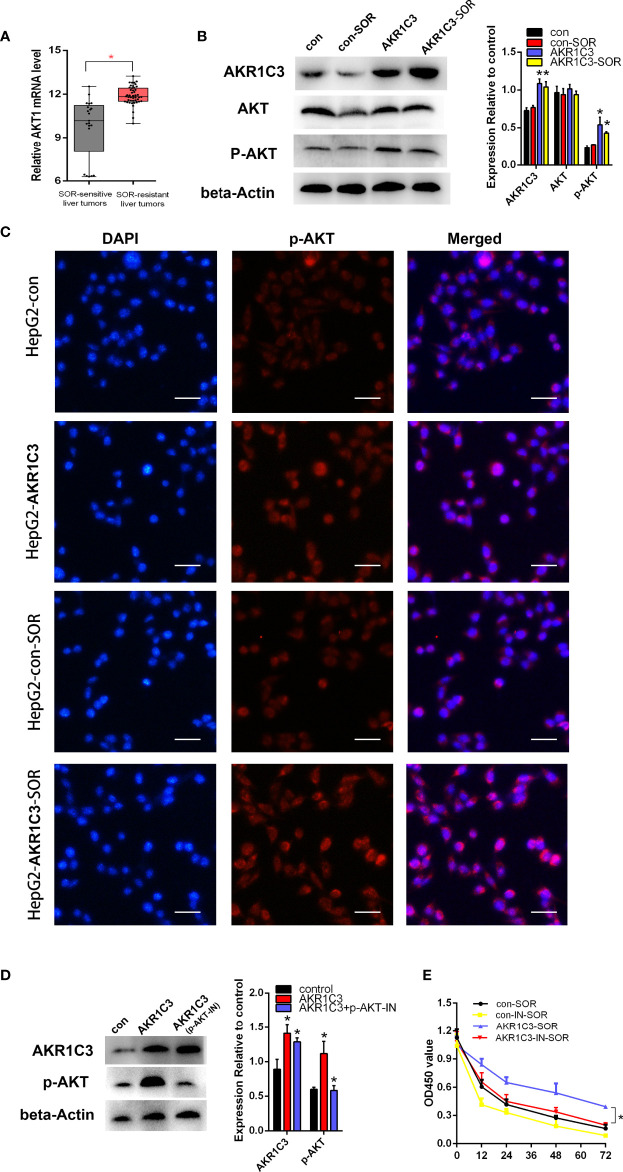
AKR1C3 promoted sorafenib resistance through AKT phosphorylation in liver cancer cells. **(A)** The mRNA expression level of AKT in sorafenib‐sensitive (*n* = 21) and sorafenib‐resistant (*n* = 46) patients. **(B)** The protein levels of AKR1C3, AKT, and p-AKT in HepG2 cells (control group and AKR1C3 overexpression group) treated with or without sorafenib (10 μM). **(C)** Immunostaining images of p-AKT in HepG2 cells (AKR1C3 overexpression group and control group) treated with sorafenib (10 μM) or without sorafenib; scale bars, 50 μm. **(D)** The efficiency of the p-AKT inhibitor was detected by Western blot. **(E)** The MTT assay of HepG2 cells (control, overexpressing AKR1C3) with or without 5 nM AZD5363 for 72 h. Data are presented as mean ± SD and are representative of three independent experiments. *P < 0.05.

Previous studies have shown that AKR1C3 promoted tumor proliferation and may be correlated with the phosphorylation of AKT (20, 21). We found that the mRNA level of AKT was upregulated obviously in sorafenib-resistant patients compared with sorafenib-sensitive patients (**Figure 6A**). To confirm the protein expression of AKT and p-AKT in liver cancer cells, we first performed a Western blot. The results indicated that there was no significant change in the expression of total AKT, when AKR1C3 was overexpressed in liver cancer cells (**Figure 6B**), while that of p-AKT was upregulated significantly in overexpressed AKR1C3 cells and in AKR1C3 overexpression cells with 10 μM sorafenib (**Figure 6B**). Immunofluorescence staining further showed that the fluorescence intensity of p-AKT was increased in overexpressed AKR1C3 cells in the nucleus and cytoplasm (**Figure 6C**). Moreover, AKR1C3 in HepG2 cells with 10 μM sorafenib can promote the expression of p-AKT (**Figure 6C**). To further explore the role of p-AKT in AKR1C3 resistance to sorafenib, we performed MTT experiments with a p-AKT inhibitor (AZD5363). The efficiency of the p-AKT inhibitor was detected by Western blot (**Figure 6D**). We treated HepG2 cells (control, overexpressing AKR1C3) with or without 5 nM AZD5363 for 72 h. All cells were treated with 10 μM sorafenib and then the MTT assay was performed. It was found that AZD5363 can significantly reduce cell proliferation caused by AKR1C3 overexpression in HepG2 cells (**Figure 6E**). Based on these findings, we concluded that AKR1C3 enhanced the resistance of sorafenib by increasing the expression of p-AKT in HCC cells.

## Discussion

Most patients with liver cancer are diagnosed when the progress is already at advanced stages or when cancer has already metastasized. During this time, surgery is difficult to achieve sufficient curative effect, and the prognosis is very poor. Sorafenib is the only drug approved by the FDA for advanced liver cancer, but due to its frequent drug resistance, it can only extend the survival period by 2.8 months, which is far from meeting the needs of the patients (22). Therefore, it is urgent to find the resistance mechanism of sorafenib to better extend the survival period of patients.

Sorafenib can induce ferroptosis and exert antitumor effects. Some studies have found that some pathways can resist ferroptosis induced by sorafenib. For example, the p62/Keap1/NRF2 pathway can directly regulate the expression of ferroptosis genes to inhibit ferroptosis caused by sorafenib in HCC (23). The oxidative stress molecules MTIG, TXNRD1, MTHFD1L, and NADPH have all been shown to be related to ferroptosis (24, 25). It is reported that FGF19/FGFR4 inhibits sorafenib-induced ROS production and apoptosis (26), and FGF19/FGFR4 is the upstream of NRF2 (27). In our experiment, a dataset of patients with sorafenib treatment was used. Based on the DEGs between different response groups, we performed GO and KEGG analyses. We discovered that oxidative stress, energy metabolism, and ferroptosis pathways were enriched in the sorafenib‐resistant group (**Figures 1E, F**). Many ferroptosis-related genes were upregulated in the sorafenib-resistant group, which may be related to the high metabolic level of tumors. Tumors with high malignancy and poor prognosis tend to have higher metabolic levels.

Current studies have proven that the AKR1C family is used as NADPH-dependent 3-, 17-, and 20-ketosteroid reductases, and different types have strong substrate specificity. AKR1C3 is mainly involved in cell proliferation and differentiation in a hormone-independent manner (28). The expression of AKR1C3, as a radioresistance-associated gene, is associated with various diseases, such as breast cancer (29), PC (30), esophageal cancer (31), and non-small cell lung cancer (NSCLC) (32). In our experiments, downregulation of AKR1C3 restrained cell proliferation and increased the sensitivity of liver cancer cells to sorafenib, and upregulation of AKR1C3 increased cell proliferation in HCC cells. Hepatocellular carcinoma displays a high degree of hypoxia (33) and expresses high levels of AKR1C3 (34). PR-104 is activated by reductases under hypoxia or by AKR1C3 to form cytotoxic nitrogen mustards. A previous study evaluated the safety and efficacy of PR-104 plus sorafenib in HCC. However, because of the compromised clearance of PR-104A and the clinically significant toxicities (thrombocytopenia mainly and neutropenia), the study was discontinued (35). Our results demonstrated that the PI3K–AKT signaling pathway was enriched in sorafenib resistance groups and overexpression of AKR1C3 in HCC cells can activate AKT. The activation of the AKT signal was often shown to be related to the treatment outcome, and it has been observed that it leads to resistance to chemotherapy and radiation therapy (36, 37). AKT phosphorylation was regulated by AKR1C3 and might be responsible for eliminating over-produced ROS in esophageal adenocarcinoma (EAC) cells (20). The intracellular ROS levels were induced by cadmium treatment. In addition, cadmium elicited the AKR1C3 expression which partially passed through the activation of PI3K (21). AKR1C3-mediated DOX resistance might result from the activation of anti-apoptosis PTEN/Akt pathway *via* PTEN loss in breast cancer (15). Overexpression of AKR1C3 to eliminate reactive oxygen species (ROS) allows the continuous activation of the AKT pathway in tumor cells upregulated by AKR1C3, thereby reducing cell apoptosis. Whether AKT is a direct target of AKR1C3 in HCC, we will further design experiments to confirm this speculation.

In summary, we found that AKR1C3 expression was induced obviously in the sorafenib-resistant group and knockdown of AKR1C3 suppressed p-Akt protein levels, ultimately leading to the decrease of HCC cell proliferation. In this respect, elucidating AKR1C3 might be a promising strategy for improving responses to sorafenib and overcoming drug resistance.

## Data Availability Statement

The datasets presented in this study can be found in online repositories. The names of the repository/repositories and accession number(s) can be found in the article/supplementary material.

## Author Contributions

ZY and JZ designed the research studies. ZY, JZ, LY, and RY contributed to the methodology. YL acquired the data. JZ, LY, and RY were responsible for the formal analysis. ZY wrote the original draft of the manuscript. ZY acquired funding. All authors contributed to the article and approved the submitted version.

## Funding

This work was financially sponsored by grants from the Tangshan Talent Funding Project A202010008.

## Conflict of Interest

The authors declare that the research was conducted in the absence of any commercial or financial relationships that could be construed as a potential conflict of interest.

## Publisher’s Note

All claims expressed in this article are solely those of the authors and do not necessarily represent those of their affiliated organizations, or those of the publisher, the editors and the reviewers. Any product that may be evaluated in this article, or claim that may be made by its manufacturer, is not guaranteed or endorsed by the publisher.

## References

[1] Jemal A Bray F Center MM Ferlay J Ward E Forman D. Global Cancer Statistics. CA: Cancer J Clin (2011) 61:69–90. doi: 10.3322/caac.20107 21296855

[2] Beste LA Leipertz SL Green PK Dominitz JA Ross D Ioannou GN. Trends in Burden of Cirrhosis and Hepatocellular Carcinoma by Underlying Liver Disease in US Veterans, 2001-2013. Gastroenterology (2015) 149:1471–82.e1475; quiz e1417–78. doi: 10.1053/j.gastro.2015.07.056 26255044

[3] Walker M El-Serag HB Sada Y Mittal S Ying J Duan Z. Cirrhosis Is Under-Recognised in Patients Subsequently Diagnosed With Hepatocellular Cancer. Aliment Pharmacol Ther (2016) 43:621–30. doi: 10.1111/apt.13505 PMC4742403 26784271

[4] Mou Y Wang J Wu J He D Zhang C Duan C. Ferroptosis, a New Form of Cell Death: Opportunities and Challenges in Cancer. J Hematol Oncol (2019) 12:34. doi: 10.1186/s13045-019-0720-y 30925886 PMC6441206

[5] Louandre C Marcq I Bouhlal H Lachaier E Godin C Saidak Z. The Retinoblastoma (Rb) Protein Regulates Ferroptosis Induced by Sorafenib in Human Hepatocellular Carcinoma Cells. Cancer Lett (2015) 356:971–7. doi: 10.1016/j.canlet.2014.11.014 25444922

[6] Nie J Lin B Zhou M Wu L Zheng T. Role of Ferroptosis in Hepatocellular Carcinoma. J Cancer Res Clin Oncol (2018) 144:2329–37. doi: 10.1007/s00432-018-2740-3 PMC11813439 30167889

[7] Endo S Oguri H Segawa J Kawai M Hu D Xia S. Development of Novel AKR1C3 Inhibitors as New Potential Treatment for Castration-Resistant Prostate Cancer. J Med Chem (2020) 63:10396–411. doi: 10.1021/acs.jmedchem.0c00939 32847363

[8] Lin HK Jez JM Schlegel BP Peehl DM Pachter JA Penning TM. Expression and Characterization of Recombinant Type 2 3 Alpha-Hydroxysteroid Dehydrogenase (HSD) From Human Prostate: Demonstration of Bifunctional 3 Alpha/17 Beta-HSD Activity and Cellular Distribution. Mol Endocrinol (1997) 11:1971–84. doi: 10.1210/mend.11.13.0026 9415401

[9] Yepuru M Wu Z Kulkarni A Yin F Barrett CM Kim J. Steroidogenic Enzyme AKR1C3 Is a Novel Androgen Receptor-Selective Coactivator That Promotes Prostate Cancer Growth. Clin Cancer Res (2013) 19:5613–25. doi: 10.1158/1078-0432.CCR-13-1151 23995860

[10] Zhao J Zhang M Liu J Liu Z Shen P Nie L. AKR1C3 Expression in Primary Lesion Rebiopsy at the Time of Metastatic Castration-Resistant Prostate Cancer Is Strongly Associated With Poor Efficacy of Abiraterone as a First-Line Therapy. Prostate (2019) 79:1553–62. doi: 10.1002/pros.23875 31294486

[11] Nakarai C Osawa K Akiyama M Matsubara N Ikeuchi H Yamano T. Expression of AKR1C3 and CNN3 as Markers for Detection of Lymph Node Metastases in Colorectal Cancer. Clin Exp Med (2015) 15:333–41. doi: 10.1007/s10238-014-0298-1 PMC4522272 24934327

[12] Heibein AD Guo B Sprowl JA Maclean DA Parissenti AM. Role of Aldo-Keto Reductases and Other Doxorubicin Pharmacokinetic Genes in Doxorubicin Resistance, DNA Binding, and Subcellular Localization. BMC Cancer (2012) 12:381. doi: 10.1186/1471-2407-12-381 22938713 PMC3495881

[13] Liu C Lou W Zhu Y Yang JC Nadiminty N Gaikwad NW. Intracrine Androgens and AKR1C3 Activation Confer Resistance to Enzalutamide in Prostate Cancer. Cancer Res (2015) 75:1413–22. doi: 10.1158/0008-5472.CAN-14-3080 PMC4383695 25649766

[14] Liu C Armstrong CM Lou W Lombard A Evans CP Gao AC. Inhibition of AKR1C3 Activation Overcomes Resistance to Abiraterone in Advanced Prostate Cancer. Mol Cancer Ther (2017) 16:35–44. doi: 10.1158/1535-7163.MCT-16-0186 27794047 PMC5222693

[15] Zhong T Xu F Xu J Liu L Chen Y. Aldo-Keto Reductase 1C3 (AKR1C3) Is Associated With the Doxorubicin Resistance in Human Breast Cancer *via* PTEN Loss. Biomed Pharmacother = Biomed Pharmacother (2015) 69:317–25. doi: 10.1016/j.biopha.2014.12.022 25661377

[16] Verma K Zang T Penning TM Trippier PC. Potent and Highly Selective Aldo-Keto Reductase 1C3 (AKR1C3) Inhibitors Act as Chemotherapeutic Potentiators in Acute Myeloid Leukemia and T-Cell Acute Lymphoblastic Leukemia. J Med Chem (2019) 62:3590–616. doi: 10.1021/acs.jmedchem.9b00090 PMC6528660 30836001

[17] Liu Y He PC Chen PC Liu PC Feng PC Liu PC. Overview of AKR1C3: Inhibitor Achievements and Disease Insights. J Med Chem (2020) 63:11305–29. doi: 10.1021/acs.jmedchem.9b02138 32463235

[18] Pinyol R Montal R Bassaganyas L Sia D Takayama T Chau GY. Molecular Predictors of Prevention of Recurrence in HCC With Sorafenib as Adjuvant Treatment and Prognostic Factors in the Phase 3 STORM Trial. Gut (2019) 68:1065–75. doi: 10.1136/gutjnl-2018-316408 PMC6580745 30108162

[19] van Malenstein H Dekervel J Verslype C Van Cutsem E Windmolders P Nevens F. Long-Term Exposure to Sorafenib of Liver Cancer Cells Induces Resistance With Epithelial-to-Mesenchymal Transition, Increased Invasion and Risk of Rebound Growth. Cancer Lett (2013) 329:74–83. doi: 10.1016/j.canlet.2012.10.021 23111106

[20] Zhou C Wang Z Li J Wu X Fan N Li D. Aldo-Keto Reductase 1C3 Mediates Chemotherapy Resistance in Esophageal Adenocarcinoma *via* ROS Detoxification. Cancers (2021) 13 (10):2403. doi: 10.3390/cancers13102403 34065695 PMC8156851

[21] Lee YJ Lee GJ Baek BJ Heo SH Won SY Im JH. Cadmium-Induced Up-Regulation of Aldo-Keto Reductase 1C3 Expression in Human Nasal Septum Carcinoma RPMI-2650 Cells: Involvement of Reactive Oxygen Species and Phosphatidylinositol 3-Kinase/Akt. Environ Toxicol Pharmacol (2011) 31:469–78. doi: 10.1016/j.etap.2011.03.006 21787718

[22] Llovet JM Ricci S Mazzaferro V Hilgard P Gane E Blanc JF. Sorafenib in Advanced Hepatocellular Carcinoma. N Engl J Med (2008) 359:378–90. doi: 10.1056/NEJMoa0708857 18650514

[23] Sun X Ou Z Chen R Niu X Chen D Kang R. Activation of the P62-Keap1-NRF2 Pathway Protects Against Ferroptosis in Hepatocellular Carcinoma Cells. Hepatology (2016) 63:173–84. doi: 10.1002/hep.28251 PMC4688087 26403645

[24] Lee D Xu IM Chiu DK Lai RK Tse AP Lan Li L. Folate Cycle Enzyme MTHFD1L Confers Metabolic Advantages in Hepatocellular Carcinoma. J Clin Invest (2017) 127:1856–72. doi: 10.1172/JCI90253 PMC5409797 28394261

[25] Lee D Xu IM Chiu DK Leibold J Tse AP Bao MH. Induction of Oxidative Stress Through Inhibition of Thioredoxin Reductase 1 Is an Effective Therapeutic Approach for Hepatocellular Carcinoma. Hepatology (2019) 69:1768–86. doi: 10.1002/hep.30467 PMC8690574 30561826

[26] Gao L Wang X Tang Y Huang S Hu CA Teng Y. FGF19/FGFR4 Signaling Contributes to the Resistance of Hepatocellular Carcinoma to Sorafenib. J Exp Clin Cancer Res (2017) 36:8. doi: 10.1186/s13046-016-0478-9 28069043 PMC5223586

[27] Teng Y Zhao H Gao L Zhang W Shull AY Shay C. FGF19 Protects Hepatocellular Carcinoma Cells Against Endoplasmic Reticulum Stress *via* Activation of FGFR4-GSK3beta-Nrf2 Signaling. Cancer Res (2017) 77:6215–25. doi: 10.1158/0008-5472.CAN-17-2039 28951455

[28] Penning TM. Aldo-Keto Reductase (AKR) 1C3 Inhibitors: A Patent Review. Expert Opin Ther Pat (2017) 27:1329–40. doi: 10.1080/13543776.2017.1379503 PMC5724044 28895472

[29] Xu D Aka JA Wang R Lin SX. 17beta-Hydroxysteroid Dehydrogenase Type 5 Is Negatively Correlated to Apoptosis Inhibitor GRP78 and Tumor-Secreted Protein PGK1, and Modulates Breast Cancer Cell Viability and Proliferation. J Steroid Biochem Mol Biol (2017) 171:270–80. doi: 10.1016/j.jsbmb.2017.04.009 28457968

[30] Sun SQ Gu X Gao XS Li Y Yu H Xiong W. Overexpression of AKR1C3 Significantly Enhances Human Prostate Cancer Cells Resistance to Radiation. Oncotarget (2016) 7:48050–8. doi: 10.18632/oncotarget.10347 PMC5216999 27385003

[31] Li X Hong X Gao X Gu X Xiong W Zhao J. Methyl Jasmonate Enhances the Radiation Sensitivity of Esophageal Carcinoma Cells by Inhibiting the 11-Ketoprostaglandin Reductase Activity of AKR1C3. Cancer Manag Res (2018) 10:3149–58. doi: 10.2147/CMAR.S166942 PMC6124458 30214307

[32] Xie L Yu J Guo W Wei L Liu Y Wang X. Aldo-Keto Reductase 1C3 may be a New Radioresistance Marker in Non-Small-Cell Lung Cancer. Cancer Gene Ther (2013) 20:260–6. doi: 10.1038/cgt.2013.15 23519145

[33] Wu XZ Xie GR Chen D. Hypoxia and Hepatocellular Carcinoma: The Therapeutic Target for Hepatocellular Carcinoma. J Gastroenterol Hepatol (2007) 22:1178–82. doi: 10.1111/j.1440-1746.2007.04997.x 17559361

[34] Guise CP Abbattista MR Singleton RS Holford SD Connolly J Dachs GU. The Bioreductive Prodrug PR-104A Is Activated Under Aerobic Conditions by Human Aldo-Keto Reductase 1C3. Cancer Res (2010) 70:1573–84. doi: 10.1158/0008-5472.CAN-09-3237 20145130

[35] Abou-Alfa GK Chan SL Lin CC Chiorean EG Holcombe RF Mulcahy MF. PR-104 Plus Sorafenib in Patients With Advanced Hepatocellular Carcinoma. Cancer Chemother Pharmacol (2011) 68:539–45. doi: 10.1007/s00280-011-1671-3 21594722

[36] Manning BD Toker A. AKT/PKB Signaling: Navigating the Network. Cell (2017) 169:381–405. doi: 10.1016/j.cell.2017.04.001 28431241 PMC5546324

[37] Revathidevi S Munirajan AK. Akt in Cancer: Mediator and More. Semin Cancer Biol (2019) 59:80–91. doi: 10.1016/j.semcancer.2019.06.002 31173856

